# Same name, different game?—How ontogeny shapes classical monocyte phenotypes

**DOI:** 10.1038/s41435-023-00248-1

**Published:** 2024-01-20

**Authors:** Lisabeth Pimenov, Azuah Lucrecia Gonzalez, Amanda C. Doran, Sylvia Knapp

**Affiliations:** 1 Research Division Infection Biology, Department of Medicine I, Medical University of Vienna, 1090 Vienna, Austria; 2 Department of Pathology, Microbiology, and Immunology, Vanderbilt University School of Medicine, Nashville, TN USA; 3 Department of Medicine, Division of Cardiovascular Medicine, Vanderbilt University Medical Center, Nashville, TN USA

**Keywords:** Monocytes and macrophages, Innate immunity

Originating primarily from progenitors in the bone marrow, monocytes are circulating mononuclear blood phagocytes, which comprise two main subsets: classical (Ly6C^high^ in mice, CD14^+^ CD16^−^ in humans) and non-classical or patrolling (Ly6C^low^ in mice, CD14^−^ CD16^+^ in humans) [1]. Classical monocytes can act as precursors to non-classical monocytes, migrate into tissues and further differentiate into monocyte-derived dendritic cells or macrophages. However, recent fate mapping and transcriptomic analyses suggested that classical monocytes themselves are heterogeneous and comprise subpopulations derived from different bone marrow progenitors [2, 3]. In this preprint (not peer-reviewed), Trzebanski et al. report novel surface markers that characterize two phenotypically distinct subsets of classical monocytes linked to their divergent bone marrow ontogeny. They furthermore demonstrate that these monocyte subsets exhibit diverging propensities to home and differentiate into tissue-resident macrophages in selected organs.

Classical monocytes represent a circulating blood cell population (10% of human leukocytes, 4% of mouse leukocytes) which is rapidly mobilized from the bone marrow to sites of infection or inflammation [1, 4]. The long-held belief was that monocytes differentiate in the bone marrow in a linear fashion from granulocyte and macrophage progenitors (GMP) to monocyte and dendritic cell (DC) progenitors (MDP) which then give rise to monocytes. This dogma was recently challenged by seminal findings proposing a binary ontogeny of monocytes from both GMP and MDP, independently [3]. Lineage tracing using single cell RNA sequencing (scRNAseq) of progenitor-derived monocyte populations confirmed that GMP produce “neutrophil-like” monocytes (NeuMo), while MDP-derived monocytes were considered “DC-like” (DCMo) [2]. However, these two classical monocyte subtypes could only be distinguished by their transcriptomic profiles since their expression of thus far known classical monocyte surface markers only differed marginally [3]. Additionally, while there is evidence that the recruitment of these two subsets varies depending on the microbial stimulus, data on functional differences as well as impact on their differentiation into tissue-resident macrophages is lacking.

To fill this knowledge gap, Trzebanski et al. segregated two distinct populations of classical monocytes using cellular indexing of transcriptomes and epitopes (CITEseq) on whole murine bone marrow. The authors found that CD62L expression (encoded by *Sell*) neatly discriminated their cell populations both on transcriptional and surface expression levels. By further analyzing those two populations, they found CD62L^+^ monocytes to be highly enriched in neutrophil related genes and termed these cells “NeuMo”. A smaller monocyte population showed reduced expression of CD62L and increased MHC-II transcripts, while still distinct from classical DC, was therefore suggested to be “DCMo”. Additionally to CD62L and MHC-II, the authors identified CD177 as a reliable surface marker for NeuMo, and CD319 for DCMo. Importantly, these two subsets could also be distinguished in peripheral blood by flow cytometry using CD177 or CD319, and validated by bulk RNA sequencing (RNAseq) analysis of sorted blood classical (Ly6C^high^) monocyte populations.

To confirm their distinctive origin from different bone marrow progenitors, Trzebanski et al. took advantage of a double reporter fate mapping mouse model (Ms4a3^Cre^:R26^LSL-tdTomato^:Cx3cr1^GFP^). Using these mice, the authors were able to discriminate GMP (tdTomato^+^) and MDP (GFP^+^) derived classical monocytes in the bone marrow, whose transcriptomes exclusively overlapped with NeuMo or DCMo, respectively. Therefore, the authors could validate that GMP give rise to CD177^+^ NeuMo, while CD319^+^ DCMo are progeny of MDP, supporting the model of binary monocyte ontogeny [2, 3]. Interestingly, the vast majority (more than 95%) of blood classical monocytes at steady state were GMP-derived, which was in line with the neutrophil-like transcriptional profile of circulating classical monocytes.

Monocytes play a crucial role in the host’s immediate defense to immunological challenge. To address how CD177^+^ NeuMo and CD319^+^ DCMo would be affected by different inflammatory stimuli, the authors challenged mice with LPS, CpG or IFNγ. After 1 day, LPS exposure led to increased proportions of CD177^+^ NeuMo in the blood compared to control mice, while CpG and IFNγ led to expansion of DCMo, confirming previous findings [3]. This difference in circulating cell numbers pointed toward different functionality, where neutrophil-like functions such as phagocytosis and NET (neutrophil extracellular trap) formation is more advantageous during bacterial infections, whereas DC-like functions such as antigen presentation are essential upon viral recognition. Indeed, using in vitro assays, the authors found NeuMo to display superior migration and NET formation capacities, while both NeuMo and DCMo could efficiently phagocytose bacteria, thus confirming their functional divergence.

A main function of monocytes in steady state and during inflammation is the replenishment of tissue-resident macrophages, which is tissue specific [1, 4–6]. To assess the ability of NeuMo and DCMo to repopulate tissue-resident macrophage niches, the authors performed adoptive transfer experiments with a 1:1 ratio of labeled NeuMo (tdTomato^+^) and DCMo (GFP^+^) given to mice depleted of endogenous bone marrow derived macrophages and monocytes (irradiated chimeras with Cx3cr1^DTR^ bone marrow). While the transferred cells were found in similar ratios in the blood 3 days after transfer, the authors reported tissue-specific repopulation abilities of NeuMo and/or DCMo after 12 or 21 days. For example, in the intestine, NeuMo and DCMo contributed equally to intestinal macrophages. Yet, DCMo preferentially resided in the lung, giving rise to lung Lyve1^-^MHC-II^high^ interstitial macrophages. In the meninges, only NeuMo but not DCMo replenished dura mater macrophages. Finally, the authors demonstrated that NeuMo specific engraftment in the dura mater depended on CD62L, since antibody-mediated blocking of CD62L diminished NeuMo colonization of the dura mater, but not the intestine.

**Fig. 1:**
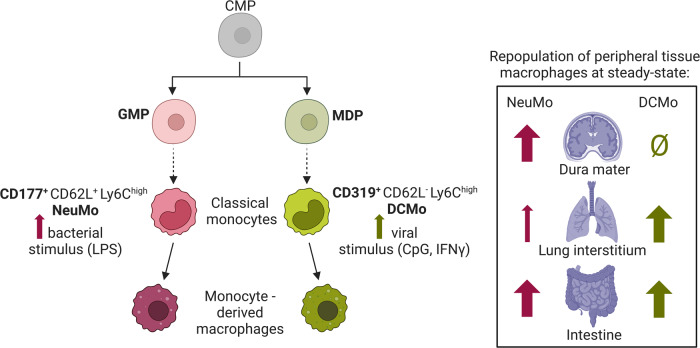
Divergent bone marrow ontogeny of classical monocytes governs distinct tissue-resident macrophage differentiation patterns. Common myeloid progenitors (CMP) give rise to two phenotypically distinct Ly6C^high^ classical monocyte subsets which derive from granulocyte-macrophage progenitors (GMP) or monocyte-DC progenitors (MDP): the GMP-derived CD177^+^ neutrophil-like monocytes (NeuMo) and the MDP-derived CD319^+^ DC-like monocytes (DCMo), which have different expansion properties in the blood depending on inflammatory stimuli (LPS, CpG, IFNγ). In addition, they display distinct homing capabilities and variable patterns of differentiation into monocyte-derived tissue macrophages at steady state, with equal NeuMo and DCMo contribution to intestinal macrophages, yet biased contributions to dura mater and lung interstitial macrophage populations. Created with BioRender.com.

Together, this study provided a novel set of surface markers to characterize murine classical monocyte subsets with neutrophil-like and DC-like phenotypes (Fig. 1, left). This will greatly facilitate future studies that address the role of these subsets in different disease contexts. Since not all classical monocytes are labeled by CD177 or CD319 and a double negative population with NeuMo-like transcriptional signatures remains in the blood, it will be relevant to investigate if the surface expression describes more transient monocyte cell states. The existence of these two functionally distinct classical monocytes raises the question as to whether non-classical monocytes also exhibit this heterogeneity. Although previous studies support the linear conversion from Ly6C^high^ classical monocytes to Ly6C^low^ non-classical monocytes, there is a debate as to whether Ly6C^low^ non-classical monocytes arise independently from a common myeloid progenitor [7]. Importantly, while neutrophil-like monocytes have been described in humans [8], it will be crucial to further identify similar phenotypes in human classical monocytes in subsequent work. Furthermore, the preprint highlights the divergent abilities of these monocyte subsets to replenish selected tissue-resident macrophage niches at steady state (Fig. 1, right), setting the stage for future work to address the origin of monocyte-derived macrophages in inflammatory and disease settings. This work by Trzebanski et al. [9] supports the idea that different disease triggers result in selective expansion of classical monocyte subsets. Considering the striking differences in monocyte-derived macrophage populations in different disease contexts, understanding the ontogeny of these cells might shed some light on the complexity.
