# Correction: Same name, different game?—How ontogeny shapes classical monocyte phenotypes

**DOI:** 10.1038/s41435-024-00269-4

**Published:** 2024-04-09

**Authors:** Lisabeth Pimenov, Azuah Lucrecia Gonzalez, Amanda C. Doran, Sylvia Knapp

**Affiliations:** 1 Research Division Infection Biology, Department of Medicine I, Medical University of Vienna, 1090 Vienna, Austria; 2 Department of Pathology, Microbiology, and Immunology, Vanderbilt University School of Medicine, Nashville, TN USA; 3 Department of Medicine, Division of Cardiovascular Medicine, Vanderbilt University Medical Center, Nashville, TN USA

**Keywords:** Monocytes and macrophages, Innate immunity

Correction to: *Genes & Immunity* 10.1038/s41435-023-00248-1, published online 20 January 2024

In this article the reference to the original bioRxiv submission was missing and should have been “https://www.biorxiv.org/content/10.1101/2023.07.29.551083v1”. The highlighted preprint has meanwhile been revised by S. Trzebanski et al., the new version can be found at https://www.biorxiv.org/content/10.1101/2023.07.29.551083v2, or Reference [9].

The original article has been corrected.

